# BLT2 Up-Regulates Interleukin-8 Production and Promotes the Invasiveness of Breast Cancer Cells

**DOI:** 10.1371/journal.pone.0049186

**Published:** 2012-11-07

**Authors:** Hyunju Kim, Jung-A Choi, Geun-Soo Park, Jae-Hong Kim

**Affiliations:** School of Life Sciences and Biotechnology, Korea University, Seoul, Korea; University of Virginia, United States of America

## Abstract

**Background:**

The elevated production of interleukin (IL)–8 is critically associated with invasiveness and metastatic potential in breast cancer cells. However, the intracellular signaling pathway responsible for up-regulation of IL-8 production in breast cancer cells has remained unclear.

**Methodology/Principal Findings:**

In this study, we report that the expression of BLT2 is markedly up-regulated in the highly aggressive human breast cancer cell lines MDA-MB-231 and MDA-MB-435 compared with MCF-10A immortalized human mammary epithelial cells, as determined by RT-PCR, real-time PCR and FACS analysis. Blockade of BLT2 with BLT2 siRNA knockdown or BLT2 inhibitor treatment downregulated IL-8 production and thereby diminished the invasiveness of aggressive breast cancer cells, analyzed by Matrigel invasion chamber assays. We further characterized the downstream signaling mechanism by which BLT2 stimulates IL-8 production and identified critical mediatory roles for the generation of reactive oxygen species (ROS) and the consequent activation of the transcription factor NF-κB. Moreover, blockade of BLT2 suppressed the formation of metastatic lung nodules by MDA-MB-231 cells in both experimental and orthotopic metastasis models.

**Conclusions/Significance:**

Taken together, our study demonstrates that a BLT2–ROS–NF-κB pathway up-regulates IL-8 production in MDA-MB-231 and MDA-MB-435 cells, thereby contributing to the invasiveness of these aggressive breast cancer cells. Our findings provide insight into the molecular mechanism of invasiveness in breast cancer.

## Introduction

Cancer invasion is a fundamental aspect of metastasis and is the major cause of death in cancer patients [1]. The acquisition of invasive capacity is required for cancer cells to invade surrounding tissues, to cross anatomic barriers, and to travel through the bloodstream or lymphatic system [2], all of which are properties of aggressive metastatic cancer cells [3]. An understanding of the molecular mechanisms that regulate the invasion process is thus key for development of therapeutic interventions to prevent tumor metastasis. Interleukin (IL)-8 has recently been suggested to promote the invasive and metastatic potential of breast cancer cells. In particular, increased levels of IL-8 were detected in breast cancer cells with a highly invasive phenotype [4], and the expression of IL-8 was found to correlate with the progression of metastasis in breast cancer cells [5], [6]. Clinical studies have also shown that the levels of IL-8 are higher in breast tumor tissue than in normal breast tissue, and an increased serum concentration of IL-8 has been suggested to be associated with advanced stages of breast cancer [7]. Despite the importance of IL-8 in cancer invasion and metastasis, however, the cellular signals required for the production of IL-8 in breast cancer cells have remained unknown.

Leukotriene B_4_ (LTB_4_) is a potent chemoattractant and pro-inflammatory lipid mediator that plays a role in the pathogenesis of several inflammatory diseases including asthma [8]. However, recent studies have also suggested that LTB_4_ and its receptors may regulate tumor progression by promoting cell proliferation and survival [9], [10], [11], [12], [13]. For instance, an increased abundance of LTB_4_ and its receptors has been observed in many types of tumor, including neuroblastoma as well as pancreatic, colon, and ovarian cancers [9], [10]. In addition, LY293111, an antagonist of the LTB_4_ receptor BLT1, inhibited the growth of and induced apoptosis in human pancreatic cancer and lymphoma cells *in vitro*. Moreover, LY293111 treatment inhibited the growth of tumor xenografts and reduced the incidence of metastasis in athymic mice [11]. Expression of the LTB_4_ receptor BLT2 in ovarian, bladder and breast cancer tissue was also found to be increased at advanced cancer stages and to be associated with a poor clinical outcome [9], [13], [14], [15]. Similarly, BLT2 was detected in infiltrating tumor cells of pancreatic adenocarcinoma tissue and in lymph node metastases [12]. Despite these various observations implicating BLT2 as a potential marker for aggressive metastatic cancer, the precise mechanism by which BLT2 promotes invasion and metastasis has not been definitively identified, especially in breast cancer cells.

We have now found that BLT2 expression is markedly increased in highly metastatic MDA-MB-231 and MDA-MB-435 human breast cancer cells and that BLT2 plays a key role in the invasiveness of these cells. We also show that reactive oxygen species (ROS) produced by NADPH oxidase 1 (Nox1) act as downstream mediators of BLT2 signaling and induce up-regulation of IL-8 through activation of nuclear factor (NF)–κB, thereby promoting the invasiveness of these cells. In addition, the formation of metastatic lung nodules by MDA-MB-231 cells was greatly suppressed by BLT2 inhibition in both experimental and orthotopic metastatic settings. Together, our results implicate a BLT2–Nox1–ROS–NF-κB cascade in up-regulation of IL-8 production and the consequent promotion of invasiveness in highly aggressive breast cancer cells. These finding will provide insight into the molecular mechanisms of invasion and metastasis in breast cancer cells.

## Results

### BLT2 Contributes to the Invasiveness of Aggressive Breast Cancer Cells

**Figure 1:**
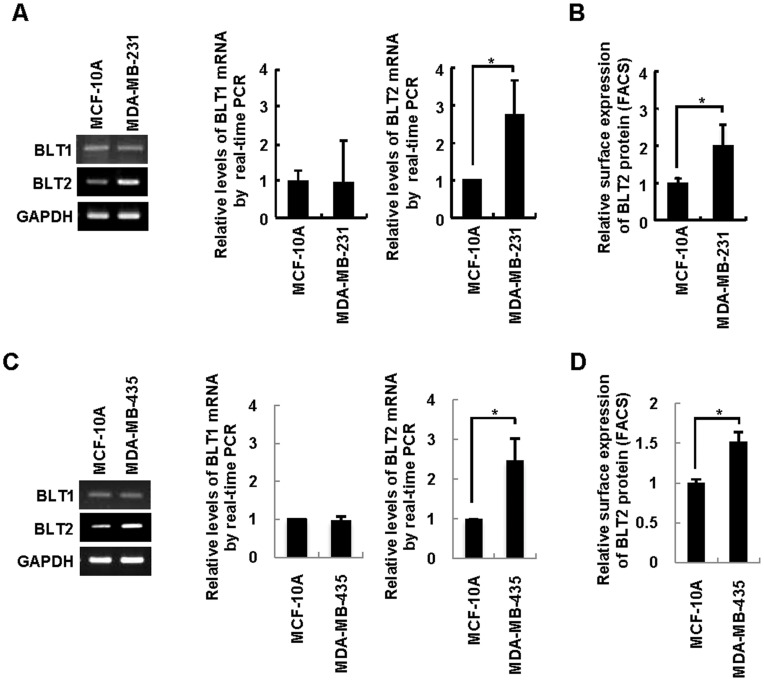
Up-regulation of BLT2 in highly invasive breast cancer cells. (A and C) Semiquantitative RT-PCR (left panel) and quantitative real-time PCR (center and right panels) analysis of BLT1 and BLT2 mRNAs in MCF-10A (control) as well as MDA-MB-231 (A) and MDA-MB-435 (C) cells. Data are representative of three independent experiments. (B and D) Flow cytometric analysis of surface BLT2 expression. All quantitative data are expressed relative to the value for MCF-10A cells and are means±SD from three independent experiments. *P<0.05.

To elucidate the role of BLT2 in the invasiveness of breast cancer, we first analyzed BLT2 expression in the highly invasive breast cancer cell lines MDA-MB-231 and MDA-MB-435, both of which have been studied extensively with regard to the characterization of metastatic processes [5], [16]. Both semiquantitative RT-PCR and quantitative real-time PCR analysis revealed that the amount of BLT2 mRNA, but not that of BLT1 mRNA, was markedly increased (about twofold) in MDA-MB-231 and MDA-MB-435 cells compared with that in immortalized mammary epithelial MCF-10A cells (Figure 1A and 1C). Consistent with these results, the abundance of BLT2 on the cell surface as determined by flow cytometry was significantly higher for MDA-MB-231 and MDA-MB-435 cells than for MCF-10A cells (Figure 1B and 1D). Together, these results indicated that BLT2 expression is markedly up-regulated in highly invasive breast cancer cells.

**Figure 2:**
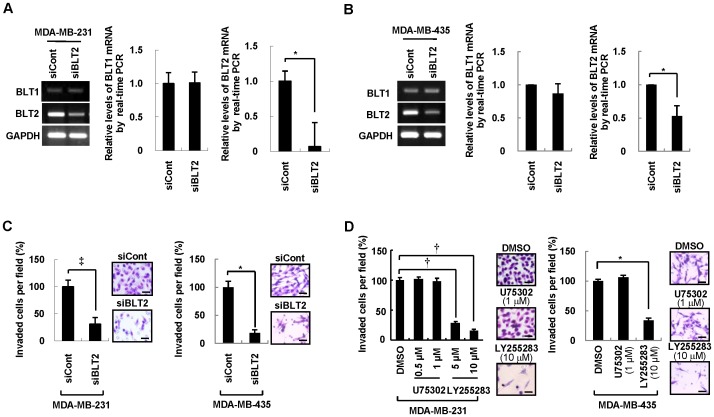
BLT2 is required for the invasiveness of MDA-MB-231 and MDA-MB-435 breast cancer cells. (A and B) MDA-MB-231 (A) and MDA-MB-435 (B) cells were transfected with BLT2 (siBLT2) or control (siCont) siRNAs for 48 h in medium supplemented with 0.5% FBS, after which the amounts of BLT1 and BLT2 mRNAs were determined by semiquantitative RT-PCR (left panel) and quantitative real-time PCR (center and right panels) analysis. Data are representative of five independent experiments. (C) Cells treated as in (A) and (B) were assayed for invasiveness. Representative invading cells stained with H&E are shown, and quantitative data are expressed relative to the value for cells transfected with the control siRNA. Scale bars, 50 µm. (D) MDA-MB-231 and MDA-MB-435 cells were incubated with U75302 (0.5 or 1 µM), LY255283 (5 or 10 µM), or DMSO vehicle for 30 min and were then assayed for invasiveness in the continued presence of the corresponding test agent. Scale bars, 50 µm. All quantitative data are means±SD from five independent experiments. *P<0.05, †*P*<0.01, ‡*P*<0.005.

We next determined the effect of BLT2 depletion by RNAi on the invasiveness of MDA-MB-231 and MDA-MB-435 cells with the use of a modified Boyden chamber assay [17]. Transfection of the cells with a BLT2 siRNA resulted in the knockdown of BLT2 mRNA but did not affect the amount of BLT1 mRNA (Figure 2A and 2B). Furthermore, BLT2 knockdown suppressed the invasive activity of MDA-MB-231 and MDA-MB-435 cells (Figure 2C). Depletion of BLT2 had no significant inhibitory effect on the proliferation (Figure S1A) or survival (Figure S1B) of these cells, suggesting that its effect on invasiveness was specific. Exposure of MDA-MB-231 and MDA-MB-435 cells to LY255283, a selective pharmacological inhibitor of BLT2 [18], [19], also attenuated cell invasiveness (Figure 2D) without affecting cell proliferation (Figure S1C) or survival (Figure S1D), whereas the BLT1 inhibitor U75302 had no such effect. Together, these results suggested that BLT2 is necessary for the invasiveness of MDA-MB-231 and MDA-MB-435 cells.

### ROS Produced by a BLT2-Nox1 Pathway are Necessary for the Invasiveness of Breast Cancer Cells

**Figure 3:**
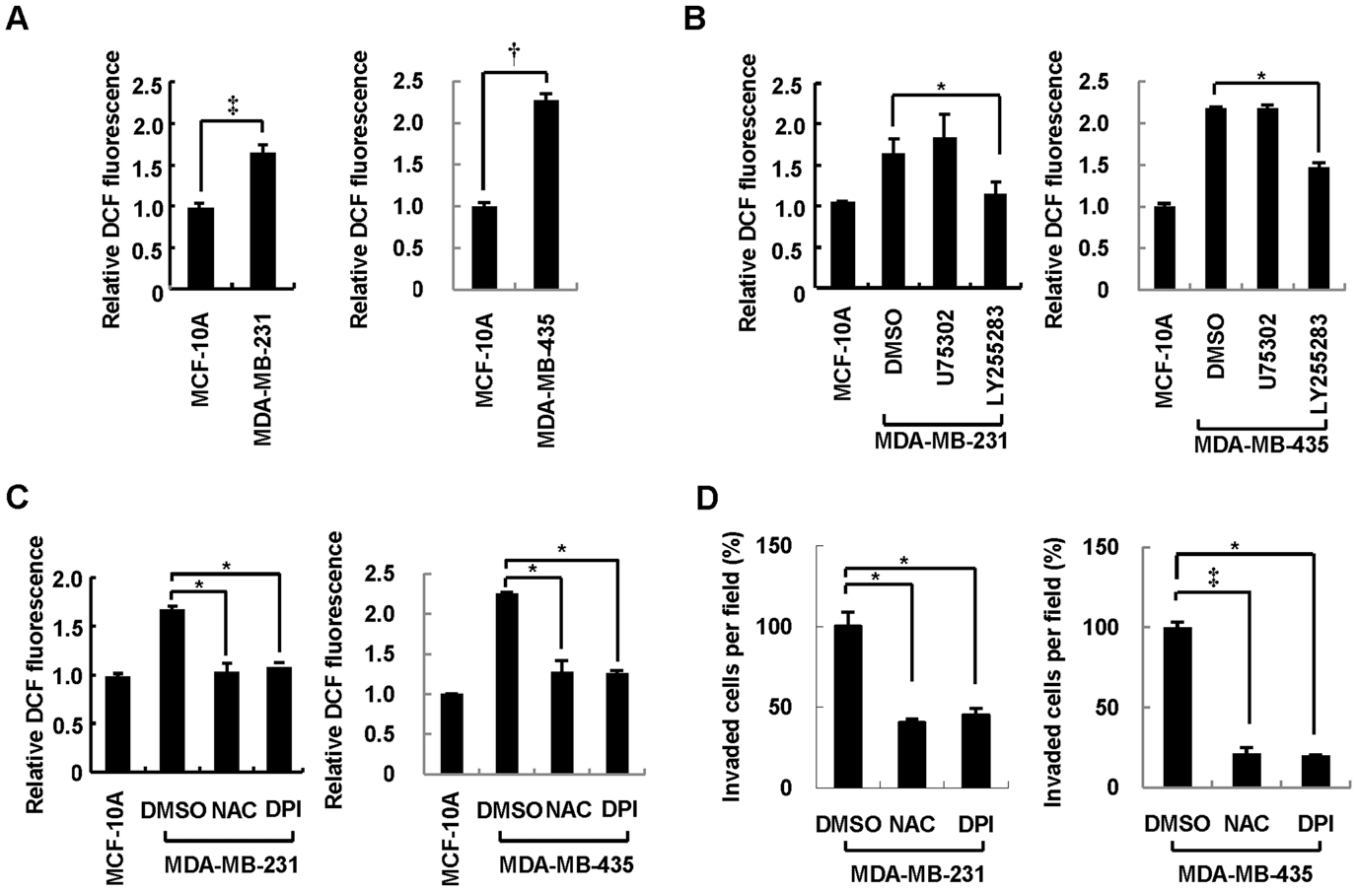
ROS production occurs downstream of BLT2 and contributes to the invasiveness of breast cancer cells. (A) MCF-10A, MDA-MB-231, and MDA-MB-435 cells were incubated in the presence of 0.5% serum for 3 h and then loaded with the diacetate form of DCF (20 µM) for 20 min. Intracellular ROS levels were then determined by flow cytometric analysis of DCF fluorescence. (B) MDA-MB-231 and MDA-MB-435 cells were incubated with U75302 (1 µM), LY255283 (10 µM), or DMSO vehicle for 1 h before determination of the intracellular level of ROS as in (A). (C and D) MDA-MB-231 and MDA-MB-435 cells were incubated with NAC (5 mM), DPI (0.5 µM), or DMSO vehicle for 1 h and then monitored for ROS levels (C) or assayed for invasiveness in the continued presence of the test agents (D). All data are expressed relative to the corresponding control value and are means±SD from five independent experiments. *P<0.05, †*P*<0.01, ‡*P*<0.005.

Our previous observations implicated ROS production downstream of BLT2 signaling in the mechanism of Ras transformation or bladder cancer progression [14], [20], [21]. We therefore next examined whether ROS contribute to the invasive phenotype of MDA-MB-231 and MDA-MB-435 cells. With the use of the redox-sensitive fluorescent probe dichlorofluorescein (DCF), we found that the intracellular level of ROS was increased in MDA-MB-231 and MDA-MB-435 cells compared with that in MCF-10A cells (Figure 3A). The increase in ROS levels apparent in MDA-MB-231 and MDA-MB-435 cells was markedly attenuated by exposure of the cells to the BLT2 inhibitor LY255283, whereas it was not affected by the BLT1 inhibitor U75302 (Figure 3B), suggesting that increased production of ROS in these cells is dependent on BLT2. Depletion of ROS by addition of the antioxidant *N*-acetylcysteine (NAC) (Figure 3C) resulted in significant inhibition of the invasiveness of MDA-MB-231 and MDA-MB-435 cells (Figure 3D), implicating the increased ROS production in the invasive phenotype of these cells. We next examined the role of Nox in this phenotype, given that we previously found that Nox catalyzes BLT2-dependent ROS production in Ras-transformed fibroblasts [21]. Treatment with DPI, an inhibitor of flavoenzymes such as Nox, largely abolished the increase in ROS levels in MDA-MB-231 and MDA-MB-435 cells (Figure 3C), suggesting that Nox may mediate ROS production in these cells. Diphenyleneiodonium (DPI) also greatly inhibited the invasiveness of MDA-MB-231 and MDA-MB-435 cells (Figure 3D). Under these experimental conditions, treatment of NAC or DPI had no inhibitory effects on cell proliferation or survival in these breast cancer cells (data not shown).

**Figure 4:**
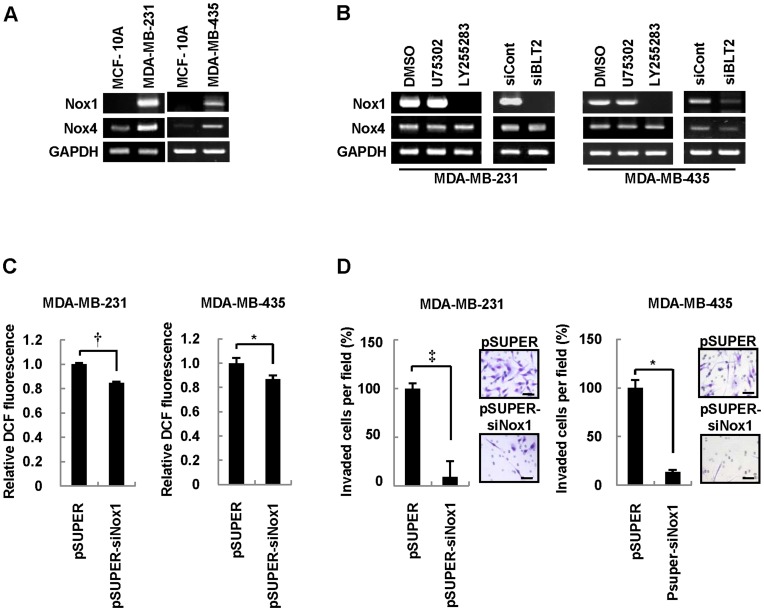
Nox1 catalyzes ROS generation associated with BLT2 signaling in MDA-MB-231 and MDA-MB-435 cells. (A) Semiquantitative RT-PCR analysis of Nox1 and Nox4 mRNAs in MCF-10A, MDA-MB-231, and MDA-MB-435 cells. Data are representative of three independent experiments. (B) MDA-MB-231 and MDA-MB-435 cells were incubated with U75302 (1 µM), LY255283 (10 µM), or DMSO vehicle for 1 h or were transfected with BLT2 or control siRNAs for 48 h before determination of the abundance of Nox1 and Nox4 mRNAs by semiquantitative RT-PCR analysis. Data are representative of three independent experiments. (C and D) MDA-MB-231 and MDA-MB-435 cells were transfected with a vector for Nox1 siRNA (pSUPER-siNox1) or the corresponding empty vector for 48 h and were then analyzed for the intracellular level of ROS (C) or invasiveness (D). Scale bars, 50 µm. Data are expressed relative to the corresponding value for cells transfected with the empty vector and are means±SD from five independent experiments. *P<0.05, †*P*<0.01, ‡*P*<0.005.

To examine the extent to which Nox activity contributes to BLT2-dependent ROS generation, we first determined the abundance of Nox mRNAs in MDA-MB-231 and MDA-MB-435 cells. The amounts of both Nox1 and Nox4 mRNAs were much higher in MDA-MB-231 and MDA-MB-435 cells than in MCF-10A cells (Figure 4A). In addition, the level of Nox1 mRNA, but not that of Nox4 mRNA, in both MDA-MB-231 and MDA-MB-435 cells was greatly reduced by exposure to LY255283, whereas U75302 had no such effect (Figure 4B). Similarly, the amount of Nox1 mRNA in both these cell lines was markedly reduced by transfection with BLT2 siRNA (Figure 4B), suggesting that Nox1 may function downstream of BLT2 in the regulation of cell invasiveness. Consistent with the proposed role of Nox1 in ROS production in MDA-MB-231 and MDA-MB-435 cells, the intracellular ROS level was significantly reduced by RNAi-mediated depletion of Nox1 (Figure 4C and S2). In addition, the invasiveness of MDA-MB-231 and MDA-MB-435 cells was significantly inhibited by transfection with Nox1 siRNA (Figure 4D). Together, these results suggested that a BLT2-Nox1-ROS pathway regulates the invasiveness of aggressive breast cancer cells.

### BLT2-ROS Signaling Activates NF-κB in MDA-MB-231 and MDA-MB-435 Cells

**Figure 5:**
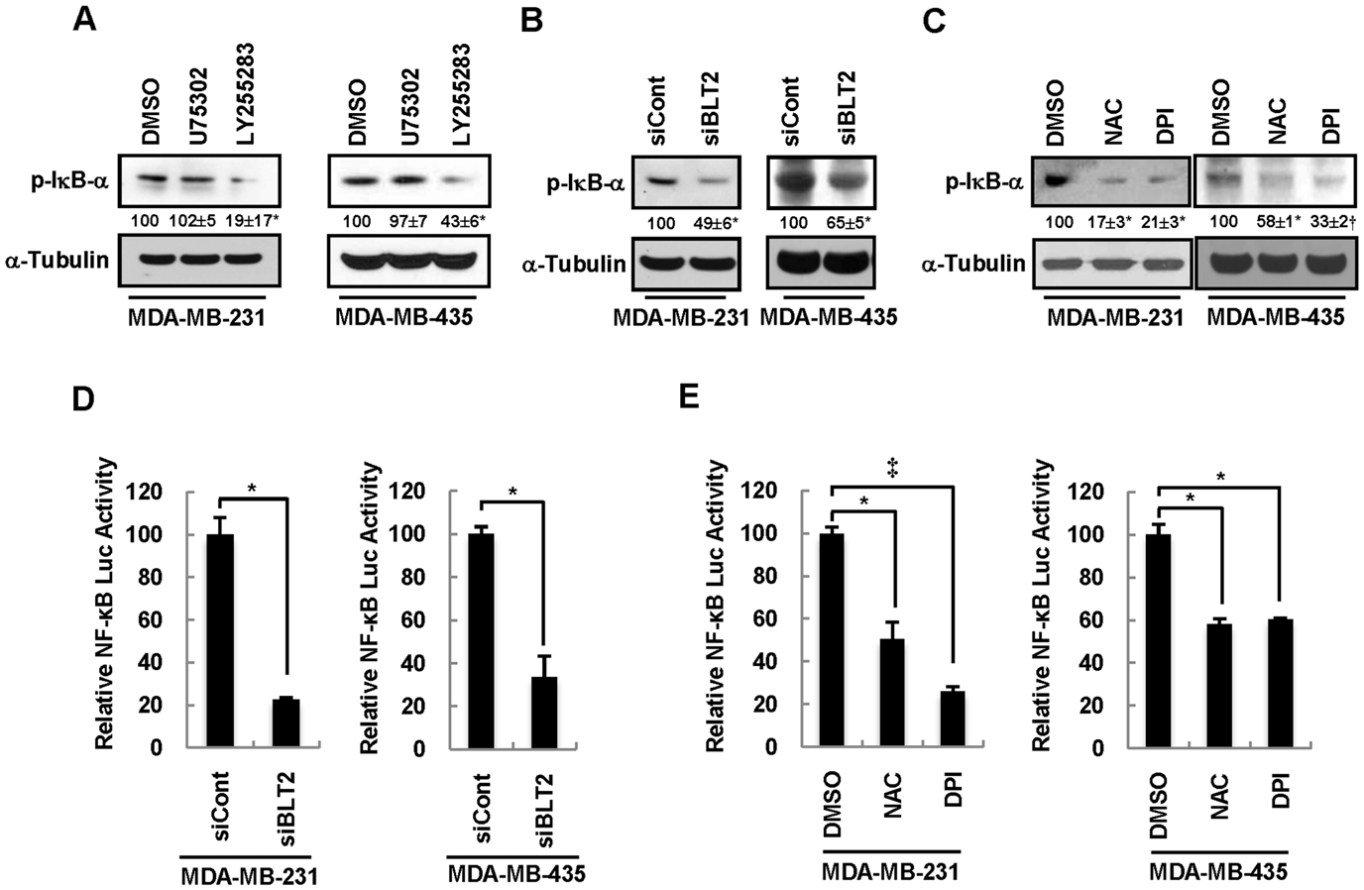
NF-κB is activated downstream of the BLT2-Nox1-ROS signaling pathway. (A–C) MDA-MB-231 and MDA-MB-435 cells were incubated with U75302 (1 µM), LY255283 (10 µM), or DMSO vehicle for 6 h (A), were transfected with BLT2 or control siRNAs for 48 h (B), or were incubated with NAC (5 mM), DPI (0.5 µM), or DMSO for 6 h (C). Cell lysates were then subjected to immunoblot analysis with antibodies to phosphorylated IκB-α (p-IκB-α) and to α-tubulin (loading control). Numbers under lanes indicate relative density of p-IκB-α from three independent experiments. (D and E) MDA-MB-231 and MDA-MB-435 cells were transiently transfected with control or BLT2 siRNAs and the pNF-κB-Luc reporter plasmid for 24 h, deprived of serum for 12 h, and then harvested (D). Alternatively, the cells were transiently transfected with pNF-κB-Luc, incubated with NAC (5 mM), DPI (0.5 µM), or DMSO for 6 h, and harvested (E). All cells were then assayed for relative luciferase activity. Data are expressed relative to the corresponding control value and are means±SD from three independent experiments. *P<0.05, †*P*<0.01, ‡*P*<0.005.

The activity of NF-κB is markedly increased in invasive breast cancer cells [16], [22], [23], suggesting that NF-κB signaling plays a key role in regulation of the invasiveness of breast cancer. Increased production of ROS has also been shown to activate NF-κB [23]. We therefore next examined whether NF-κB might function downstream of BLT2-ROS signaling in regulation of the invasiveness of MDA-MB-231 and MDA-MB-435 cells. Immunoblot analysis revealed that LY255283, but not U75302, markedly inhibited the phosphorylation of the endogenous NF-κB inhibitor IκB-α (Figure 5A), whose phosphorylation by the IκB kinase complex results in NF-κB activation [24]. Depletion of BLT2 by RNAi also inhibited the phosphorylation of IκB-α in these cells (Figure 5B). To examine whether ROS contribute to NF-κB activation, we determined the effects of the Nox inhibitor DPI and the ROS scavenger NAC. Both NAC and DPI inhibited IκB-α phosphorylation in MDA-MB-231 and MDA-MB-435 cells (Figure 5C). Consistent with these results, the activity of an NF-κB–dependent luciferase reporter gene was also significantly inhibited by BLT2 depletion (Figure 5D) as well as by NAC or DPI (Figure 5E). Together, these results suggested that NF-κB activation occurs downstream of BLT2-ROS signaling in MDA-MB-231 and MDA-MB-435 cells.

### BLT2–ROS–NF-κB Signaling Induces IL-8 Expression in MDA-MB-231 and MDA-MB-435 Cells

**Figure 6:**
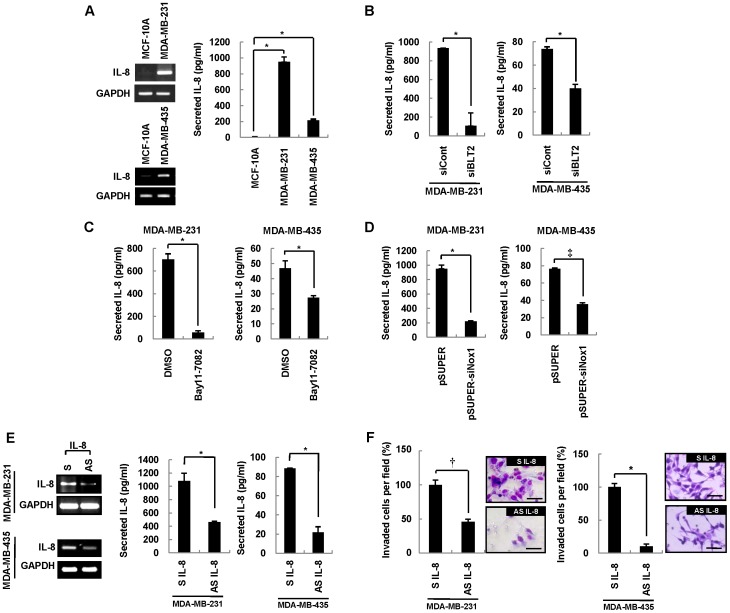
BLT2-dependent production of IL-8 contributes to the invasiveness of breast cancer cells. (A) MCF-10A, MDA-MB-231, and MDA-MB-435 cells were subjected to semiquantitative RT-PCR analysis of IL-8 mRNA (left panels). The cells (2×10^5^) per well in six-well plates were also incubated for 48 h, after which the amount of IL-8 released into the medium was measured with the use of a specific ELISA (right panel). (B) MDA-MB-231 and MDA-MB-435 cells were transfected with BLT2 or control siRNAs for 48 h, after which the secretion of IL-8 was determined as in (A). (C) MDA-MB-231 and MDA-MB-435 cells were incubated with Bay11-7082 (10 µM) or DMSO vehicle for 48 h, after which the amount of IL-8 released into the medium was determined. (D) MDA-MB-231 and MDA-MB-435 cells were transfected with a vector for Nox1 siRNA (pSUPER-siNox1) or the corresponding empty vector for 48 h, after which the secretion of IL-8 was determined as in (A). (E and F) MDA-MB-231 and MDA-MB-435 cells were transfected with sense (S) or antisense (AS) IL-8 oligonucleotides for 48 h, after which the amounts of IL-8 were determined by semiquantitative RT-PCR and ELISA assays (E) and the cells were assayed for invasiveness (F). Scale bars, 50 µm. Semiquantitative RT-PCR data are representative of three independent experiments and all quantitative data are means±SD from three independent experiments. *P<0.05, †*P*<0.01, ‡*P*<0.005.

IL-8 expression is associated with the invasiveness and metastatic potential of aggressive breast cancer cells [23], [25]. We therefore examined the possible role of IL-8 in regulation of the invasiveness of MDA-MB-231 and MDA-MB-435 cells by the BLT2 signaling cascade. The amounts of IL-8 mRNA and protein were markedly increased in MDA-MB-231 and MDA-MB-435 cells compared with control MCF-10A cells (Figure 6A). Furthermore, the abundance of IL-8 mRNA in MDA-MB-231 and MDA-MB-435 cells was markedly reduced by exposure to LY255283, whereas it was little affected by treatment with U75302 (Figure S3A). In addition, depletion of BLT2 by RNAi resulted in a similar marked reduction in the amounts of IL-8 mRNA (Figure S3A) and protein (Figure 6B) in these cells, indicating that BLT2 signaling up-regulates IL-8 expression. Among the BLT2 ligands tested, we observed that only LTB_4_ could enhance the levels of IL-8 (Figure S4A) and invasiveness (Figure S4C) in MDA-MB-231 and MDA-MB-435 cells. In addition, LTB_4_ synthesis inhibitor MK886 suppressed the levels of IL-8 (Figure S4B) and invasiveness (Figure S4D), while 12-lipoxygenase inhibitor (baicalein) and thromboxane synthesis inhibitor (OKY-046) had no effect (data not shown). Together, we propose that a LTB_4_-BLT2 signaling pathway is likely to up-regulate IL-8 expression and thereby increase the invasiveness of breast cancer cells. To evaluate further the role of the BLT2–ROS–NF-κB cascade in the up-regulation of IL-8, we examined the effects of Bay11-7082, a specific inhibitor of IκB kinase. Inhibition of NF-κB signaling by this agent resulted in down-regulation of IL-8 mRNA (Figure S3B) and protein (Figure 6C) in MDA-MB-231 and MDA-MB-435 cells. Similarly, treatment with DPI or NAC markedly reduced the amount of IL-8 mRNA in these cells (Figure S3C). In addition, knockdown of Nox1 resulted in depletion of IL-8 mRNA (Figure S3C) and protein (Figure 6D). These observations thus suggested that the BLT2–Nox1–ROS–NF-κB cascade up-regulates IL-8 expression in highly invasive breast cancer cells. To evaluate whether this BLT2-dependent IL-8 expression contributes to the invasiveness of MDA-MB-231 and MDA-MB-435 cells, we examined the effect of an IL-8 antisense oligonucleotide. The marked decrease in the abundance of IL-8 mRNA and protein induced by transfection of MDA-MB-231 and MDA-MB-435 cells with this antisense oligonucleotide (Figure 6E) was accompanied by significant inhibition of cell invasiveness (Figure 6F) but no effect on cell growth (Figure S3D), suggesting that IL-8 indeed contributes to the invasive phenotype of these cells. Collectively, these results indicated that the BLT2–ROS–NF-κB cascade is a previously unrecognized regulator of IL-8 production, and that such regulation controls the invasiveness of highly aggressive breast cancer cells.

### Role of BLT2 in Metastasis of Breast Cancer Cells in vivo

**Figure 7:**
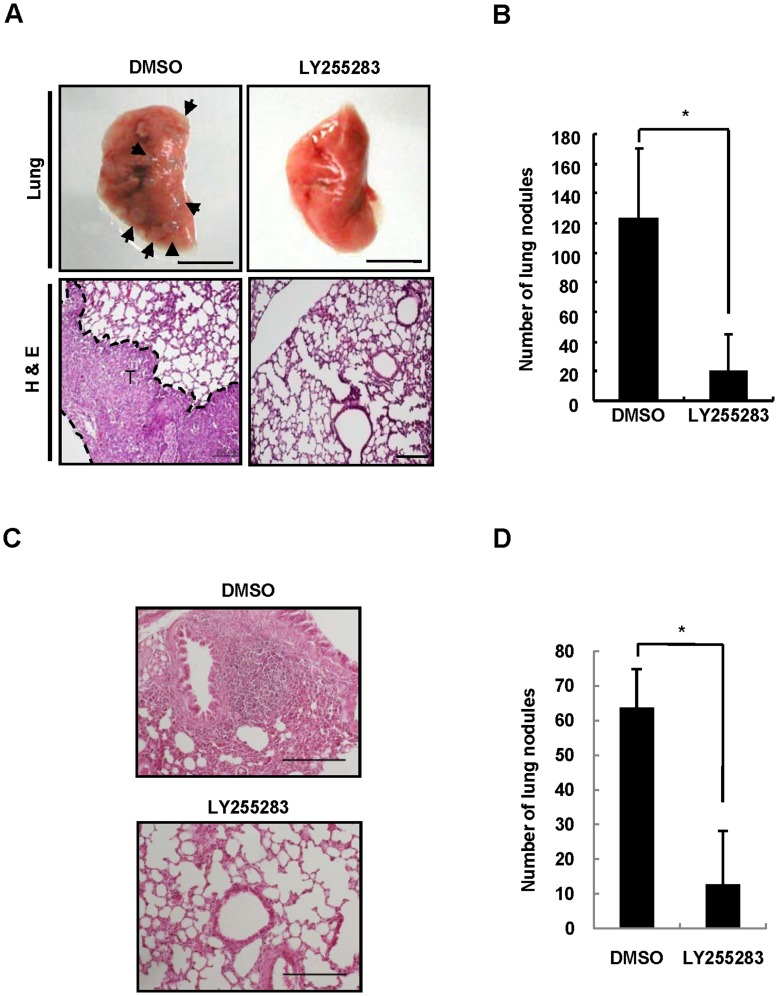
BLT2 inhibition suppresses metastasis of MDA-MB-231 cells *in vivo*. (A and B) MDA-MB-231 cells treated for 24 h with LY255283 (10 µM) or DMSO were injected into the tail vein of nude mice, which were subsequently injected intraperitoneally with DMSO (control) or LY255283 (2.5 mg/kg) at 3 and 5 days after cell injection. Mice were killed 10 weeks after cell injection, and the lungs were removed and subjected to histological analysis (A). Arrowheads indicate metastatic nodules. Scale bars, 5 mm (upper panels) and 200 µm (lower panels). The number of metastatic nodules in the lungs was also counted (B); data are means±SD of values from four mice of each group. *P<0.05. (C and D) MDA-MB-231 cells pretreated for 24 h with LY255283 (10 µM) or DMSO were implanted into the mammary fat pad of nude mice, and the animals were then injected intraperitoneally with LY255283 (2.5 mg/kg) or DMSO (control) three times at 5-day intervals. The mice were killed 14 weeks after cell implantation, and lung sections were stained with H&E for analysis of metastatic nodules (C). Scale bars, 200 µm. The number of metastatic nodules in the lung was also counted (D). Data are representative of results obtained with three mice per group. *P<0.05.

Cell invasiveness is closely associated with metastatic potential [3]. We therefore finally examined the role of BLT2 in the metastatic potential of MDA-MB-231 cells *in vivo*. To this end, we first investigated the effect of LY255283 in an *in vivo* experimental metastasis assay in which cancer cells are injected into the tail vein of nude mice [26]. MDA-MB-231 cells were pretreated with 10 µM LY255283 or DMSO for 24 h prior to tail vein injection. Mice injected with MDA-MB-231 cells and treated with DMSO vehicle manifested large metastatic nodules in the lungs, whereas the number of such nodules was reduced by ∼80% by treatment with LY255283 (Figure 7A and 7B). We also examined the effect of LY255283 on the metastatic potential of aggressive breast cancer cells in an orthotopic metastasis assay in which MDA-MB-231 cells were implanted into the mammary fat pad. MDA-MB-231 cells pretreated for 24 h with 10 µM LY255283 or DMSO were implanted into the mammary fat pad of mice, and the animals were then administrated intraperitoneally with LY255283 or DMSO three times at 5-day intervals. Administration of LY255283 resulted in a marked reduction in the extent of metastasis to the lungs compared with that apparent in mice treated with DMSO (Figure 7C and 7D). Together, these results implicated BLT2 in metastasis of highly aggressive MDA-MB-231 breast cancer cells *in vivo*.

## Discussion

In the present study, we have found that the expression level of BLT2 is markedly increased in the highly aggressive breast cancer cell lines MDA-MB-231 and MDA-MB-435. Selective attenuation of BLT2 function by LY255283 treatment or RNAi-mediated knockdown resulted in suppression of the invasiveness of MDA-MB-231 and MDA-MB-435 cells, implicating BLT2 as a key determinant of the invasive phenotype of breast cancer. In addition, we characterized the BLT2 signaling pathway responsible for the regulation of cell invasiveness, finding that the Nox1-dependent generation of ROS and NF-κB activation function downstream of BLT2 to induce up-regulation of IL-8, a cytokine previously implicated in breast cancer invasiveness and metastasis. Finally, we showed that the formation of metastatic lung nodules by MDA-MB-231 cells was greatly suppressed by BLT2 blockade in both experimental and orthotopic metastasis models. Together, our findings suggest that a BLT2–Nox1–ROS–NF-κB signaling cascade up-regulates IL-8 expression and thereby contributes to the invasiveness of aggressive breast cancer cells.

BLT2 is a G protein–coupled receptor that is expressed on the cell surface and interacts with specific ligands such as LTB_4_, 12(*S*)-HETE and 12-HHT [18], [19], [27]. BLT1, another LTB_4_ receptor, is expressed mainly on inflammatory cells, such as leukocytes, and plays a role in inflammatory processes [8]. Unlike BLT1, BLT2 has a low affinity for LTB_4_ and is expressed in a wide variety of tissues. Whereas various inflammatory functions of BLT1 have been extensively characterized, few biological functions of BLT2 have been identified, although recent studies have suggested that it plays a role in inflammatory diseases [8] and cancer progression [9], [10], [11], [12], [13], [14], [20]. Consistent with our findings, the level of IL-8, which is the most effective chemotactic factor known for neutrophils [28], is highly correlated with the invasiveness of breast cancer, with increased amounts of IL-8 having been detected in breast tumors with a high invasive potential [4]. High serum concentrations of IL-8 have also been associated with advanced stages of cancer [29]. The IL-8 produced by cancer cells is thought to act in an autocrine or paracrine manner through interaction with its cell surface receptors to promote invasiveness. Indeed, several lines of evidence have shown that the autocrine action of IL-8 drives the invasiveness of cancer cells [30], [31].

In the present study, we found that a BLT2 signaling pathway regulates the invasive phenotype of MDA-MB-231 and MDA-MB-435 breast cancer cells. However, we cannot exclude the possibility that activation of this BLT2–Nox1–ROS–NF-κB–IL-8 cascade also contributes to other cancer-promoting activities such as angiogenesis, especially *in vivo*. IL-8 also functions as an angiogenic factor, and BLT2-dependent IL-8 production may thus contribute to angiogenesis in the tumor microenvironment and thereby promote cancer progression. In addition to its potential role in angiogenesis, BLT2-ROS signaling was recently shown to promote survival in estrogen receptor (ER)–negative, nonmetastatic breast cancer cells, such as MDA-MB-468 and MDA-MB-453 cells, although the downstream effectors of this BLT2-ROS pathway were not determined [13]. However, we did not detect a role for BLT2 in the survival of MDA-MB-231 and MDA-MB-435 cells (Figure S1), which are ER-negative and highly aggressive, suggesting that BLT2 regulates the invasiveness but not the survival of these cells. The biological functions of BLT2 signaling may therefore differ even among cells that are ER-negative and may vary with breast cancer stage. We previously observed that BLT2 levels were higher in ER-positive cells such as MCF-7 than in MDA-MB-231 ER-negative cells [13]. Nonetheless, MCF-7 cells are considered less invasive. And, we speculate that the biological function of BLT2 may differ among breast cancer cells. For instance, we have observed that the proliferation, not the invasiveness, of MCF-7 cells was severely attenuated by BLT2 inhibition, pointing to BLT2 as a mediator of the proliferation, not the invasiveness, in MCF-7 cells (data not shown). Indeed, many G protein–coupled receptors implicated in tumorigenesis have been shown to have pleiotropic effects in this process [32]. Further studies will be required to elucidate the potential pleiotropic actions of BLT2 in cancer progression, including its roles in the various stages of breast cancer development.

**Figure 8:**
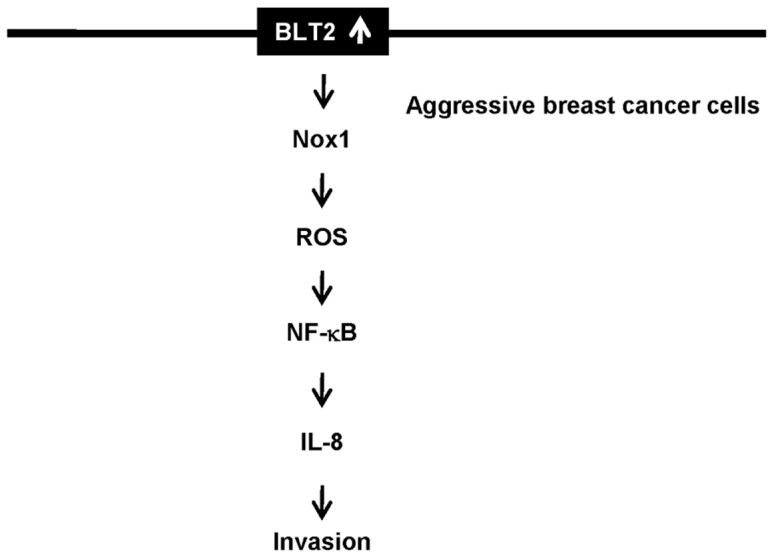
Scheme for the involvement of BLT2 cascade in IL-8 production and invasiveness of breast cancer cell. BLT2-induced IL-8 up-regulation leads to enhanced invasiveness of breast cancer cells. The activation of the BLT2 pathway induced Nox1-dependent ROS generation. Then, these ROS may contribute to NF-κB activation, which lead to IL-8-dependent invasiveness.

At the moment, the exact mechanism by which IL-8 trigger invasiveness is poorly understood in breast cancer cells. Previously, however, it was shown that up-regulation of matrix metalloproteinases (MMPs), especially MMP-2 and MMP-9, is involved in IL-8-induced invasiveness [31]. Moreover, we recently observed that MMPs are indeed downstream components of BLT2 signaling, potentially mediating the invasiveness and metastasis of ovarian and bladder cancer cells [14], [15]. Thus, we speculate that MMPs may act downstream of BLT2-IL-8 cascade in MDA-MB-231 and MDA-MB-435 cells, thereby mediating the invasiveness. A detailed mechanism for the potential link between IL-8 and MMPs remains to be further characterized. As summarized in Figure 8, we have shown that a BLT2–Nox1–ROS–NF-κB signaling cascade up-regulates the production of IL-8 in MDA-MB-231 and MDA-MB-435 cells and thereby contributes to the invasiveness and metastasis of these aggressive breast cancer cells. Our findings have thus revealed a previously unrecognized role for BLT2 in the invasiveness of aggressive breast cancer cells, and they should both contribute to a better understanding of the molecular mechanisms of breast cancer progression as well as provide potential targets for the development of new therapeutics for this condition.

## Materials and Methods

### Materials

Fetal bovine serum (FBS), horse serum, Dulbecco’s modified Eagle’s medium–F12 mixture (DMEM/F12), RPMI 1640, and nonessential amino acids were obtained from Life Technologies (Gaithersburg, MD). LTB_4_, 12(*S*)-hydroxyeicosatetraenoic acid (12(*S*)-HETE), 12(*S*)-hydroxyheptadeca-5Z, 8E, 10E-trienoic acid (12-HHT), MK886, baicalein, OKY-046, U75302 and LY255283 were from Cayman Chemical (Ann Arbor, MI). Bovine serum albumin, dimethyl sulfoxide (DMSO), cholera enterotoxin, hydrocortisone, recombinant human epidermal growth factor, bovine insulin, diphenylene iodonium, and *N*-acetylcysteine were from Sigma-Aldrich (St. Louis, MO). Bay11-7082 and Bay11-7085 were from Calbiochem (La Jolla, CA). All other chemicals were obtained from standard sources and were of molecular biology grade or higher.

### Cell Culture

The human breast cancer cell lines MDA-MB-231 and MDA-MB-435 were obtained from the Korean Cell Line Bank (Seoul, Korea) and Jackson Laboratory (Bar Harbor, ME), respectively. These cells were maintained in RPMI 1640 supplemented with 10% heat-inactivated FBS, 2 mM glutamine, penicillin (100 U/ml), and streptomycin (100 U/ml). Human immortalized mammary epithelial MCF-10A cells were kindly provided by A. Moon (Duksung Women’s University, Seoul, Korea) [33] and were maintained in DMEM/F12 supplemented with 5% heat-inactivated horse serum, insulin (10 µg/ml), epidermal growth factor (20 ng/ml), cholera enterotoxin (100 ng/ml), hydrocortisone (0.5 µg/ml), penicillin (100 U/ml), and streptomycin (100 U/ml). All cells were maintained at 37°C under an atmosphere of 5% CO_2_.

### Semiquantitative RT-PCR Analysis of BLT1, BLT2, Nox1, Nox4, and IL-8 mRNAs

Total RNA was extracted from cells with the use of Easy Blue (Intron, Sungnam, Korea), dissolved in diethylpyrocarbonate-treated water, and quantified by measurement of absorbance at 260 nm. The RNA (1.25 µg) was subjected to reverse transcription (RT) followed by polymerase chain reaction (PCR) amplification of BLT2, Nox4, IL-8, and glyceraldehyde-3-phosphate dehydrogenase (GAPDH) cDNAs with the use of an RT-PCR PreMix Kit (Intron), as described previously [34]. PCR amplification of BLT1 cDNA was performed with the primers 5′-TATGTCTGCGGAGTCAGCATGTACGC-3′ (sense) and 5′-CCTGTAGCCGACGCCCTATGTCCG-3′ (antisense). Amplification was confirmed to be in the linear range of the reaction. The amplification protocol for BLT1 cDNA included 30 cycles of denaturation at 95°C for 30 s, annealing at 67°C for 20 s, and elongation at 72°C for 40 s. Analysis of Nox1 mRNA was performed by RT and a two-step PCR procedure as described previously [35]. RT was thus performed with the reverse primer of the first-round PCR (see below), and cDNA derived from 1.25 µg of total RNA was subjected to the first-round PCR with the forward Nox1 primer 5′-CAGGGAGACAGGTGCCTTTTCC-3′ (beginning at nucleotide 1523, GenBank accession no. NM 007052) and the reverse Nox1 primer 5′-GCTCAAACCTGACGAGACCAAG-3′ (ending at nucleotide 2037). For the second-round, nested PCR, the forward nested primer was 5′-AACCTGTTGACTTCCCTGGAAC-3′ (beginning at nucleotide 1554) and the reverse nested primer was 5′-TCCAGACTGGAATATCGGTGAC-3′ (ending at nucleotide 1858). The amplification protocol for both first-round and nested PCR included 27 cycles of denaturation at 95°C for 30 s, annealing at 61°C for 40 s, and elongation at 72°C for 45 s. The specificity of all primers was confirmed by sequence analysis of the PCR products.

### RT and Real-time Quantitative PCR Analysis of BLT1 and BLT2 mRNAs

Total RNA (1.25 µg) extracted from cells with the use of Easy Blue (Intron) was subjected to RT with Moloney murine leukemia virus reverse transcriptase (Invitrogen, Carlsbad, CA), and BLT1, BLT2, and GAPDH cDNAs were amplified as described previously [13] with the use of a LightCycler 480 SYBR Green I Master instrument (Roche, Mannhein, Germany). Melting curves were analyzed to ensure amplification specificity for the PCR products. The amounts of BLT1 and BLT2 mRNAs were normalized by the corresponding amount of GAPDH mRNA.

### Flow Cytometric Analysis of BLT2

For quantitation of BLT2 expression on the cell surface, cells were incubated in 60-mm dishes for 24 h. The cells were detached by treatment with trypsin, washed with PBS and fixed in 2% paraformaldehyde. After exposure to 2% bovine serum albumin for 30 min, the cells were incubated for 1 h at room temperature with rabbit polyclonal antibodies to BLT2 (MBL-2097, 1∶100 dilution; Life Span, Des Plaines, IL), washed three times with phosphate-buffered saline (PBS), and incubated for 30 min at room temperature with fluorescein isothiocyanate–conjugated goat antibodies to rabbit immunoglobulin G (1∶200 dilution; Molecular Probes, Eugene, OR). The cells (10,000 per sample) were then subjected to flow cytometry with the use of a FACSCalibur instrument and Cell Quest software (Becton Dickinson, Franklin Lakes, NJ) for determination of mean fluorescence intensity.

### Cell Growth Assay

Cells were plated at a density of 0.5×10^5^ or 2×10^5^ cells per well in 12-well plates and cultured for 24 h. Cells were transfected with control or BLT2 siRNAs for 24 h, after which they were incubated in medium supplemented with 0.5% FBS and incubated for the indicated times. Also, cultured cells were incubated in medium supplemented with 0.5% FBS for 3 h and then exposed to U75302 (0.5 or 1 µM) or LY255283 (5 or 10 µM) and incubated at 37°C for the indicated times. The cells were then isolated by exposure to trypsin-EDTA, and the number of viable cells was determined by staining with trypan blue and light microscopy.

### Detection of Apoptosis via FITC-Annexin V/PI Saining

Cells were plated at a density of 3×10^5^ cells per well in 6-well plates and cultured for 24 h. The cells were transfected with control or BLT2 siRNAs for 24 h, after which they were incubated in medium supplemented with 0.5% FBS and incubated for 48 h. Cells (1×10^5^) were collected and suspended in 100 µl 1×Annexin V Binding Buffer (BD Bioscience, San Jose, CA). 2 µl FITC-Annexin V (BD Bioscience) were added as well as 10 µl propidium iodide (PI) (Sigma, Saint Louis, MO) staining to a final concentration of 5 µg/ml and the cells were incubated at room temperature for 15 min in the dark. Then, 400 µl of Annexin V binding buffer were added and flow cytometry was performed using a BD FACSCalibur flow cytometer. Cells were considered to be apoptotic if they were Annexin V+/PI– (early apoptotic) and Annexin V+/PI+ (late apoptotic). Each analysis was performed using at least 20,000 events. Four independent experiments were performed.

### Flow Cytometric Analysis of Sub-G_1_ Population

The sub-G_1_ (apoptotic) population was assessed as described [36]. Cells treated as described for the cell growth assay were fixed in 70% ethanol, resuspended in 0.5 ml of PBS, mixed with 0.5 ml of DNA extraction buffer (19.2 mM Na_2_HPO_4_, 0.004% Triton X-100), and incubated for 5 min at room temperature. The cells were then isolated by centrifugation at 400×*g* for 5 min, stained for 30 min with PI (50 µg/ml) in PBS containing RNase A (100 µg/ml), and analyzed by flow cytometry with a FACScan instrument and CellQuest software (Becton Dickinson). Cells with a DNA content less than that of cells in G_1_ phase (sub-G_1_) were assumed to be apoptotic.

### RNA Interference (RNAi)

Depletion of BLT2 mRNA by RNAi was performed as described previously [13]. BLT2-specific and scrambled control small interfering RNAs (siRNAs) were designed as described previously [12] and were obtained from Ambion (Austin, TX). RNAi for Nox1 was performed by transfection of cells with pSUPER vectors (SUPPLIER) as described previously [37].

### Invasion Assays

The invasion potential of MDA-MB-231 and MDA-MB-435 cells was assessed with the use of BioCoat Matrigel Invasion Chambers (BD Biosciences, Bedford, MA). Cells (5×10^4^) were harvested from culture plates by exposure to trypsin-EDTA, washed with RPMI 1640 supplemented with 0.5% FBS, and seeded in the same medium on the rehydrated Matrigel inserts. RPMI 1640 supplemented with 5% FBS was then added to the lower chamber as a chemoattractant, and the cells were incubated at 37°C for 36 h. Cells on the filters were subsequently fixed in methanol and stained with hematoxylin-eosin (H&E). Those on the upper surface of each filter were removed, and the invading cells were then counted in 10 random high-power (×20) fields with the use of a CKX41 microscope (Olympus, Tokyo, Japan) equipped with a DP71 digital camera (Olympus). Each sample was assayed in triplicate, and the assays were performed five times.

### Measurement of ROS

Measurement of intracellular H_2_O_2_ was performed as described previously [21]. The mean fluorescence intensity of dichlorofluorescein was measured by flow cytometry with a FACSCalibur instrument (excitation, 488 nm; emission, 530 nm).

### Luciferase Reporter Assay for NF-κB Activity

MDA-MB-231 and MDA-MB-435 cells were transfected with 1.4 µg NF-κB leuciferase reporter gene [38] using Lipofectamine reagent (Invitrogen, Carlsbad, CA), according to the manufacturer’s instructions. To monitor variations in cell numbers and transfection efficiency, MDA-MB-231 and MDA-MB-435 cells were co-transfected with 0.6 µg pSV40-β-galactosidase, a eukaryotic expression vector containing the *Escherichia coli* β-galactosidase (lacZ) structural gene under the control of the SV40 promoter. After 24 h incubation in complete media, cells were incubated for 12 h in RPMI containing 0.5% FBS. The cells were then harvested and assayed for luciferase activity as described [38].

### Transfection with an Antisense Oligonucleotide for IL-8

Oligonucleotides were obtained from Bioneer (Daejeon, Korea). An antisense oligonucleotide for IL-8 was designed as previously described [39]. Cells were plated at a density of 4×10^5^ per 60-mm dish, cultured for 24 h, and transfected for 36 h with 100 nM of antisense or control sense oligonucleotides with the use of Lipofectamine (Invitrogen). The depletion of IL-8 mRNA in the cells was assessed by RT-PCR analysis.

### Immunoblot Analysis

MDA-MB-231 and MDA-MB-435 cells were collected and lysed in lysis buffer [40 mM Tris-HCl (pH 8.0), 120 mM NaCl, 0.1% Nonidet-P40, 100 mM phenylmethylsulfonyl fluoride, 1 mM sodium orthovanadate, 2 µg/ml leupeptin, 2 µg/ml aprotinin]. Proteins in the lysate were separated by SDS-PAGE and transferred onto a nitrocellulose membrane, after which the membrane was blocked with 5% nonfat dry milk in Tris-buffered saline and then incubated with primary antibodies (Santa Cruz Biotechnology, Santa Cruz, CA) against p-IκB-α and α-tubulin for 1 h at room temperature. Blots were developed with a peroxidase-conjugated secondary antibody, and proteins were visualized using ECL reagents (Amersham, Arlington Heights, IL) according to the manufacturer’s recommendations.

### ELISA for IL-8 Measurement

An enzyme-linked immunosorbent assay (ELISA) kit for human IL-8 was obtained from BD Biosciences.

### In vivo Metastasis Assays

The study was approved by the ethics committee of Korea University, and all experimental animals used in this study were treated according to the guidelines approved by the Institutional Animal Care and Use Committee of Korea University. Female nude mice (Charles River, Wilmington, MA) were used for metastasis assays and were 6 weeks old at the time of cell injection. For experimental metastasis assays, cultured MDA-MB-231 cells (5×10^5^) were pretreated with LY255283 or DMSO for 24 h to ensure the inhibition of BLT2 signaling and were then suspended in PBS and injected into the tail vein. LY255283 (2.5 mg/kg) or DMSO vehicle was injected intraperitoneally 3 and 5 days after cell injection. The mice (*n* = 4 for each group) were maintained under aseptic barrier conditions until they were killed at 10 weeks after cell injection for detection of pulmonary metastases. The number of lung surface metastasis nodules with a diameter of >0.2 mm was determined. For spontaneous metastasis assays, mice (*n* = 3 for each group) were anesthetized by intraperitoneal injection of zoletil (50 mg/kg) and xylazine (2.5 mg/kg), and then MDA-MB-231 cells (2×10^6^) pretreated with LY255283 or DMSO for 24 h in PBS were implanted into the mammary fat pad. LY255283 (2.5 mg/kg) or DMSO vehicle was injected intraperitoneally three times at 5-day intervals beginning immediately after cell implantation. The mice were killed and subjected to necropsy at 14 weeks after cell implantation. The primary tumors were removed, and the presence of lung metastases was determined by microscopic analysis. The lung and tumor tissue were fixed in 4% formalin, embedded in paraffin, sectioned, and stained with H&E for examination with a BX51 microscope (Olympus) equipped with a DP71 digital camera (Olympus).

### Data Analysis and Statistics

The results are presented as mean±SD. Analyses were performed with the Student *t* test using SigmaPlot 8.0. Values of *p*<0.05 were considered to be significant.

## Supporting Information

Figure S1 **Blockade of BLT2 with LY255283 or RNAi does not affect the proliferation or survival.** (A and B) Cells transfected with control or BLT2 siRNAs for 24 h were incubated either for the indicated times for determination of cell growth by trypan blue staining (A) or for 48 h for determination of cell survival by flow cytometric analysis of the FITC-Annexin V/PI staining and the sub-G_1_ population (B). (C and D) Cells exposed to U75302 (0.5 or 1 µM), LY255283 (5 or 10 µM), or DMSO vehicle for the indicated times (C) or for 48 h (D) were assayed for cell growth and apoptosis, respectively. All data are means±SD from three independent experiments.
(TIF)

Figure S2 **Knockdown of Nox1 by RNAi.** MDA-MB-231 and MDA-MB-435 cells were transfected with a vector for Nox1 siRNA (pSUPER-siNox1) or the corresponding empty vector for 48 h, after which total RNA was isolated from the cells and the amount of Nox1 mRNA was determined by semiquantitative RT-PCR analysis. Data are representative of three independent experiments.
(TIF)

Figure S3 **BLT2 regulates the production of IL-8.** (A) MDA-MB-231 and MDA-MB-435 cells were incubated with U75302 (1 µM), LY255283 (10 µM), or DMSO vehicle for 48 h or were transfected with BLT2 or control siRNAs for 48 h, after which the amount of IL-8 mRNA were assessed by semiquantitative RT-PCR analysis. (B) MDA-MB-231 and MDA-MB-435 cells were incubated with a specific IκB kinase inhibitor Bay11-7082 (10 µM), a specific NF-κB inhibitor Bay11-7085 (1 µM), or DMSO vehicle for 48 h and then assayed for IL-8 mRNA by semiquantitative RT-PCR analysis. (C) MDA-MB-231 and MDA-MB-435 cells were incubated with DPI (0.5 µM), NAC (5 mM), or DMSO vehicle for 48 h or were transfected with a vector for Nox1 siRNA (pSUPER-siNox1) or the corresponding empty vector for 48 h, after which the abundance of IL-8 mRNA was determined by semiquantitative RT-PCR analysis. Semiquantitative RT-PCR data are representative of three independent experiments. (D) Cells transfected with sense (S) or antisence (AS) IL-8 oligonucleotides for 24 h were incubated for the incubated times before determining cell growth by trypan blue staining. Data are means±SD from three independent experiments.
(TIFF)

Figure S4 **The addition of LTB_4_ induces the invasiveness.** (A and B) MDA-MB-231 and MDA-MB-435 cells were incubated for 48 h with LTB_4_ (50, 100, 300 or 300 nM) (A) or MK886 (5 µM) (B). Thereafter, the levels of IL-8 were assessed for mRNA and protein by semiquantitative RT-PCR (left panel) and ELISA (right panel), respectively. Data are representative of three independent experiments. (C and D) MDA-MB-231 and MDA-MB-435 cells were incubated with LTB_4_ (300 nM) (C) or MK886 (5 µM) (D) for 30 min and then assayed for the invasiveness. Scale bars, 50 µm. All quantitative data are mean±SD from five independent experiments. *P<0.05, †*P*<0.01, ‡*P*<0.005.
(TIF)
