# Supplementary figures and images for: BLT2 Up-Regulates Interleukin-8 Production and Promotes the Invasiveness of Breast Cancer Cells
Source: PLoS One. 2012 Nov 7;7(11):e49186. doi: 10.1371/journal.pone.0049186 (PMC3492316; doi:10.1371/journal.pone.0049186)

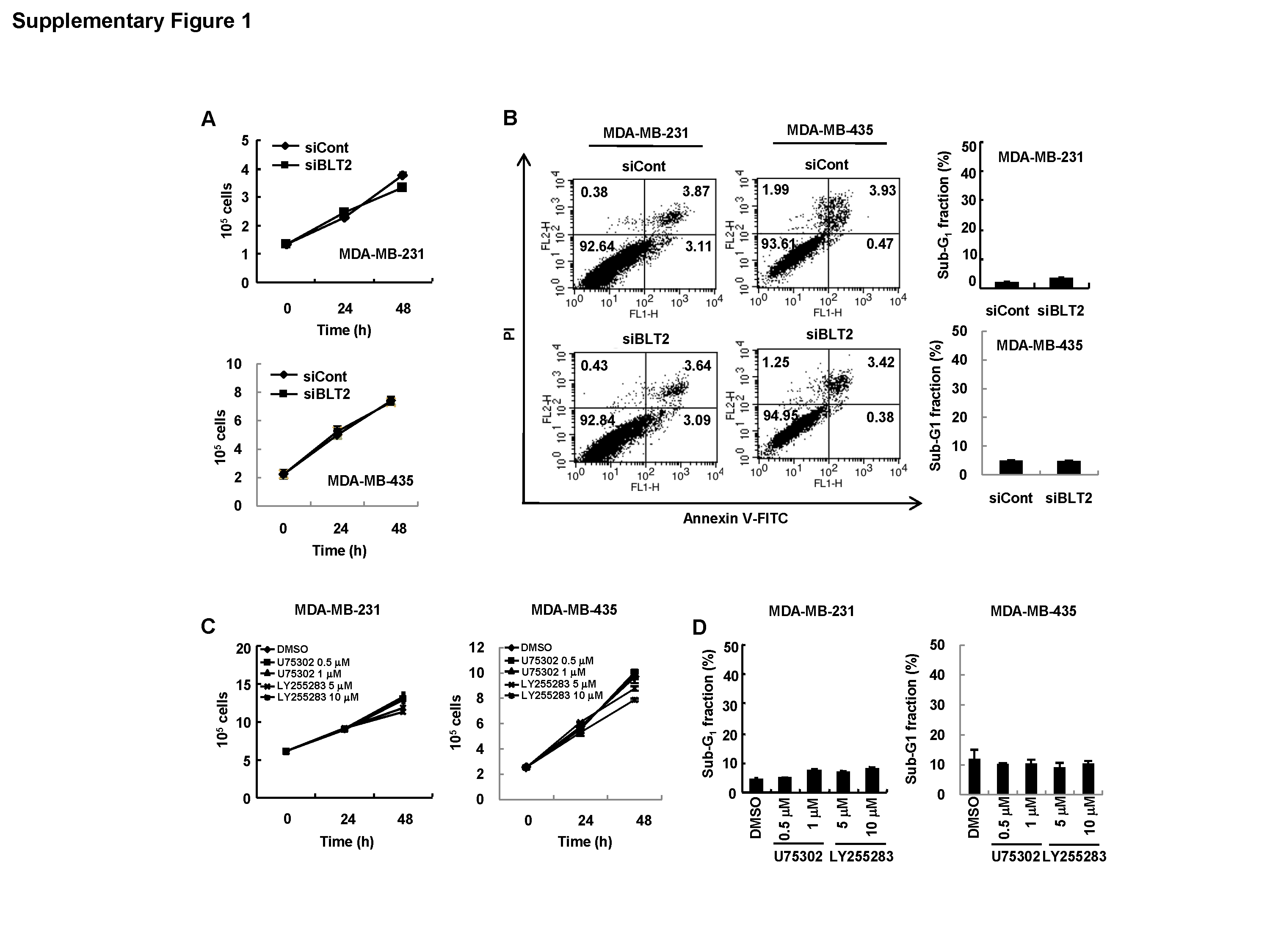

Supplement: Figure S1 — Blockade of BLT2 with LY255283 or RNAi does not affect the proliferation or survival. (A and B) Cells transfected with control or BLT2 siRNAs for 24 h were incubated either for the indicated times for determination of cell growth by trypan blue staining (A) or for 48 h for determination of cell survival by flow cytometric analysis of the FITC-Annexin V/PI staining and the sub-G1 population (B). (C and D) Cells exposed to U75302 (0.5 or 1 µM), LY255283 (5 or 10 µM), or DMSO vehicle for the indicated times (C) or for 48 h (D) were assayed for cell growth and apoptosis, respectively. All data are means±SD from three independent experiments. (TIF) [file pone.0049186.s001.tif]

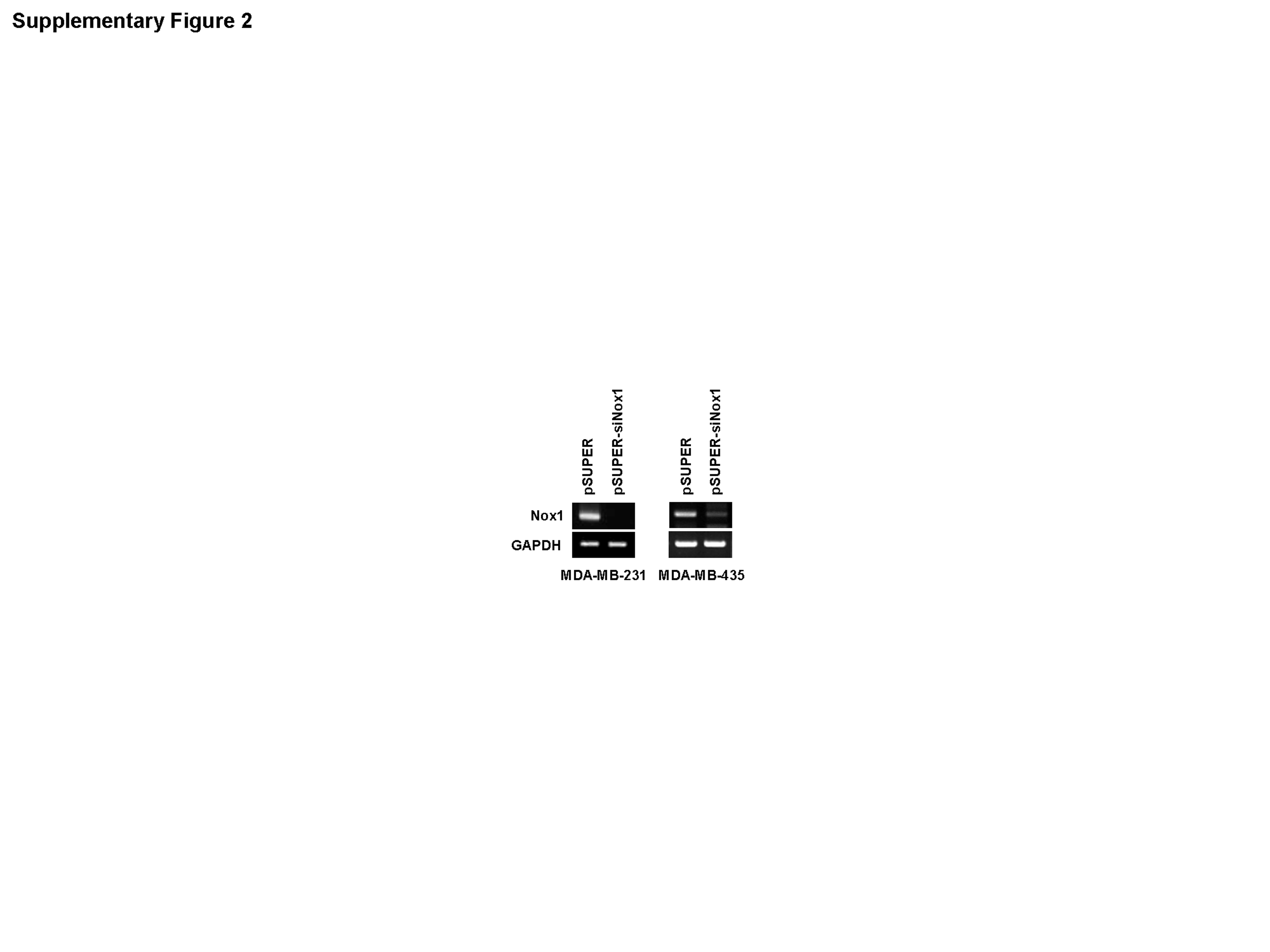

Supplement: Figure S2 — Knockdown of Nox1 by RNAi. MDA-MB-231 and MDA-MB-435 cells were transfected with a vector for Nox1 siRNA (pSUPER-siNox1) or the corresponding empty vector for 48 h, after which total RNA was isolated from the cells and the amount of Nox1 mRNA was determined by semiquantitative RT-PCR analysis. Data are representative of three independent experiments. (TIF) [file pone.0049186.s002.tif]

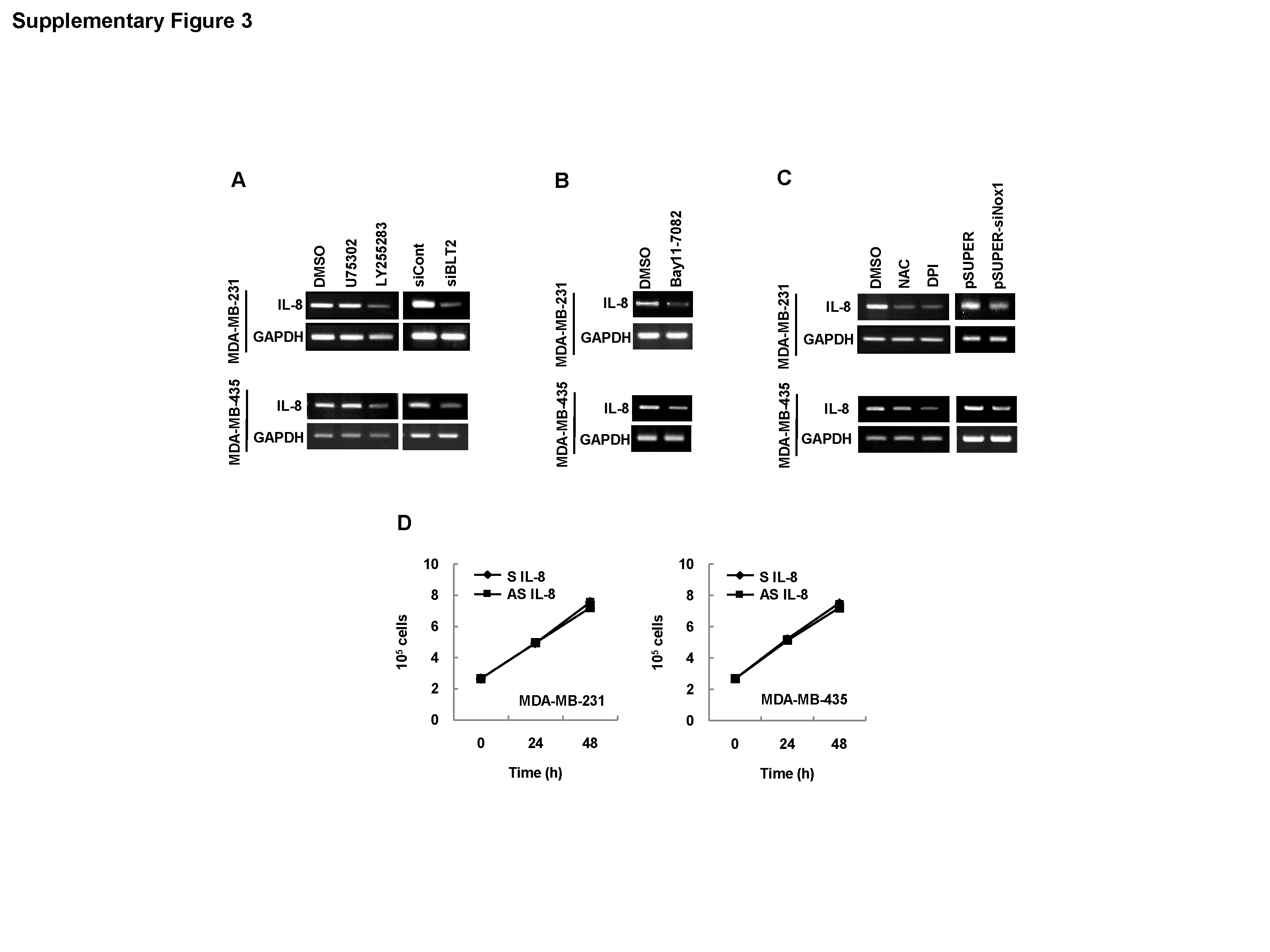

Supplement: Figure S3 — BLT2 regulates the production of IL-8. (A) MDA-MB-231 and MDA-MB-435 cells were incubated with U75302 (1 µM), LY255283 (10 µM), or DMSO vehicle for 48 h or were transfected with BLT2 or control siRNAs for 48 h, after which the amount of IL-8 mRNA were assessed by semiquantitative RT-PCR analysis. (B) MDA-MB-231 and MDA-MB-435 cells were incubated with a specific IκB kinase inhibitor Bay11-7082 (10 µM), a specific NF-κB inhibitor Bay11-7085 (1 µM), or DMSO vehicle for 48 h and then assayed for IL-8 mRNA by semiquantitative RT-PCR analysis. (C) MDA-MB-231 and MDA-MB-435 cells were incubated with DPI (0.5 µM), NAC (5 mM), or DMSO vehicle for 48 h or were transfected with a vector for Nox1 siRNA (pSUPER-siNox1) or the corresponding empty vector for 48 h, after which the abundance of IL-8 mRNA was determined by semiquantitative RT-PCR analysis. Semiquantitative RT-PCR data are representative of three independent experiments. (D) Cells transfected with sense (S) or antisence (AS) IL-8 oligonucleotides for 24 h were incubated for the incubated times before determining cell growth by trypan blue staining. Data are means±SD from three independent experiments. (TIFF) [file pone.0049186.s003.tif]

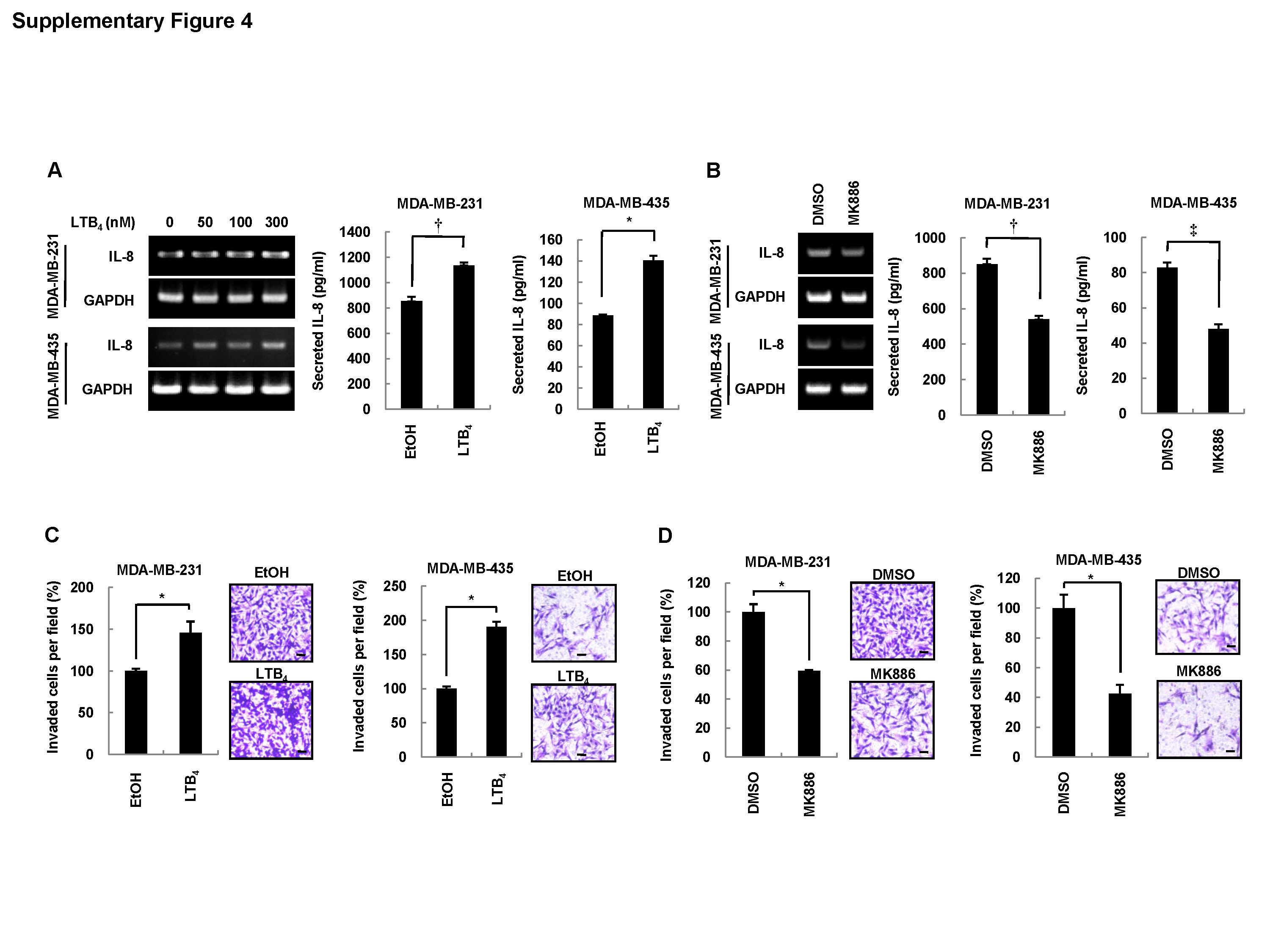

Supplement: Figure S4 — The addition of LTB4 induces the invasiveness. (A and B) MDA-MB-231 and MDA-MB-435 cells were incubated for 48 h with LTB4 (50, 100, 300 or 300 nM) (A) or MK886 (5 µM) (B). Thereafter, the levels of IL-8 were assessed for mRNA and protein by semiquantitative RT-PCR (left panel) and ELISA (right panel), respectively. Data are representative of three independent experiments. (C and D) MDA-MB-231 and MDA-MB-435 cells were incubated with LTB4 (300 nM) (C) or MK886 (5 µM) (D) for 30 min and then assayed for the invasiveness. Scale bars, 50 µm. All quantitative data are mean±SD from five independent experiments. *P<0.05, †P<0.01, ‡P<0.005. (TIF) [file pone.0049186.s004.tif]
